# Phenotype of VCP Mutations in Chinese Amyotrophic Lateral Sclerosis Patients

**DOI:** 10.3389/fneur.2022.790082

**Published:** 2022-02-07

**Authors:** Shu-Yan Feng, Han Lin, Chun-Hui Che, Hua-Pin Huang, Chang-Yun Liu, Zhang-Yu Zou

**Affiliations:** ^1^Department of Neurophysiology, Henan Provincial People's Hospital, Zhengzhou, China; ^2^Zhengzhou University People's Hospital, Zhengzhou, China; ^3^Department of Neurology, Fujian Medical University Union Hospital, Fuzhou, China; ^4^Institute of Clinical Neurology, Fujian Medical University, Fuzhou, China

**Keywords:** amyotrophic lateral sclerosis, Paget's disease of bone, valosin-containing protein, R155C, phenotype

## Abstract

Mutations in the valosin-containing protein (VCP) gene have been linked to amyotrophic lateral sclerosis (ALS) in the Caucasian populations. However, the phenotype of VCP mutations in Chinese patients with (ALS) remains unclear. Targeted next-generation sequencing covered 28 ALS-related genes including the VCP gene was undertaken to screen in a Chinese cohort of 275 sporadic ALS cases and 15 familial ALS pedigrees. An extensive literature review was performed to identify all patients with ALS carrying VCP mutations previously reported. The clinical characteristics and genetic features of ALS patients with VCP mutations were reviewed. One known p.R155C mutation in the VCP gene was detected in two siblings from a familial ALS pedigree and two sporadic individuals. In addition, the same VCP p.R155C mutation was detected in an additional patient with ALS referred in 2021. Three patients with VCP p.R155C mutation presented with muscular weakness starting from proximal extremities to distal extremities. The other patient developed a phenotype of Paget's disease of bone in addition to the progressive muscular atrophy. We reported the first VCP mutation carrier manifesting ALS with Paget's disease of bone in the Chinese population. Our findings expand the phenotypic spectrum of the VCP mutations in Chinese patients with ALS and suggest that ALS patients with VCP p.R155C mutations tend to present with relatively young onset, symmetrical involvement of proximal muscles weakness of arms or legs, and then progressed to distal muscles of limbs.

## Introduction

Amyotrophic lateral sclerosis (ALS) is a neurodegenerative disorder characterized by motor dysfunction in limbs (muscle weakness, atrophy, and spasticity) and bulbar palsy, such as dysarthria and dysphagia. Cognitive decline or behavioral impairment occurs in some cases. Approximate two-thirds of patients with ALS had a limb onset. The typical disease course of ALS cases is aggressive, ending in death due to respiratory failure within 3–5 years (1).

Approximately 5–10% of ALS cases present in a familial pattern, while the others have no family history. The genetic background of ALS is complicated, correlated with a growing spectrum of genes, such as C9orf72, SOD1, FUS, and TDP-43 (1). Valosin-containing protein (VCP) gene codes a highly conserved triple A-adenosine triphosphatase (triple A-ATPase), which operates as a regulatory factor in the procedure of endoplasmic reticulum-associated degradation (2). When the triple A-ATPase has functional deficits, the ubiquitin-dependent recycling or degradation by the proteasome will be disrupted. Additionally, the process of membrane fusion, transcriptional activation, and apoptosis heavily relies on the VCP-coded ATPase (3). Mutations in the VCP gene have been determined as a causative gene of the syndrome, inclusion body myopathy (IBM) with Paget's disease of bone (PDB), and frontotemporal dementia (FTD) (IBMPFD) since 2001 (4). The clinical spectrum of VCP gene-related diseases was expanded to ALS when a four-generation Italian ALS pedigree with VCP mutation was detected by whole-exome sequencing in 2010 (5). Subsequent studies in Caucasian populations detected VCP mutations in both familial and sporadic patients with ALS (5, 6). Mutations in the VCP gene in patients with ALS of Chinese origin have been rarely reported (7). Here, we reported the phenotype of four patients with ALS carrying VCP mutation of Chinese origin.

## Materials and Methods

### Subjects

In total, a cohort of 275 sporadic ALS cases and 15 familial ALS pedigrees was recruited at Fujian Medical University Union Hospital and Henan Provincial People's Hospital between January 2017 and December 2018. Another sporadic ALS case referred to Fujian Medical University Union Hospital in 2021 was included. Diagnosis of ALS was made according to the revised El Escorial criteria (8). Familial ALS was diagnosed if one or more first- or second-degree relatives developed ALS. The study was approved by the Ethics Committee of Fujian Medical University Union Hospital and Henan Provincial People's Hospital. All subjects involved in this research have offered written consent.

### Genetic Studies

Genomic DNA extracted from venous peripheral blood lymphocytes of both sporadic cases and the proband of the family were subjected to the targeted next-generation sequencing on Illumina Hiseq sequencer (Illumina Inc., San Diego, CA, USA). An ALS-specific gene panel which included 28 genes (SOD1, FUS, TARDBP, VCP, VAPB, SPG11, OPTN, PFN1, ANG, ALS2, DAO, UBQLN2, SIGMAR1, SETX, FIG4, DCTN1, TUBA4A, TBK1, SQSTM1, CHCHD10, MATR3, HNRNPA1, HNRNPA2B1, KIF5A, ANXA11, TIA1, CCNF, and NEK1) was designed. The targeted regions were designed to include all exons and flanking regions of the 29 genes which contained the VCP gene (NM_007126.5). The GGGGCC expansions in C9orf72 were screened as previously described (9).

As a result of sequencing, the mean on-target coverage was 880 × with an average percentage of targets covered greater or equal to 100 × of 100%. Variant filtering process has been described (10). The identified variants were subsequently validated by Sanger sequencing. Bioinformatic analysis of the variants was performed as previously described (10).

### Literature Review

We conducted a literature search in Medline to identify previous studies that screened for VCP mutations in patients with ALS. The following keywords were used: “Valosin-containing protein” OR “VCP”, in combination with “amyotrophic lateral sclerosis” OR “ALS” OR “motor neuron(e) disease” OR “MND”. Only English language literature was included in the review. For each eligible publication, the following information was extracted: name of the **first** author, publication year, population, the sample size of familial ALS (FALS), and/or sporadic ALS (SALS), numbers of VCP mutation carriers in FALS and/or SALS. For patients with ALS carrying VCP mutations, the following information was extracted from the relevant papers: **first** author, year of study, population, sex, age, family history, clinical features, and genetic characteristics.

### Statistical Analysis

In each study, the mutation frequencies of the VCP gene were reported as the number of the mutation carriers among all cases of FALS or SALS screened. Single case reports, such as the additional VCP p.R155C mutated ALS referred to our center in 2021 were not included in the meta-analysis. The mutation frequencies in different populations were combined using a fixed-effects model. A statistical analysis was carried out using the Meta function of R (R version 3.64) (https://www.r-project.org/).

## Results

### Clinical Features of the ALS Cohort

Between January 2017 and December 2018, 275 sporadic cases and 15 familial pedigrees meeting the diagnostic criteria of ALS were enrolled in our study. There were 173 men and 117 women with mean onset age of 55.3 years (SD, 11.6). Of the total cases, 19.3% of patients reported a bulbar onset, 79.0% had a limb onset, and 1.7% had a respiratory onset.

### Genetic Analysis

**Figure 1:**
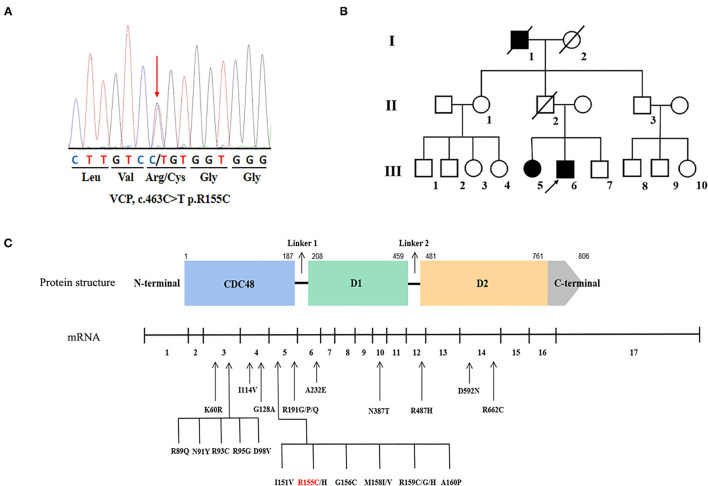
Genetic information of the valosin-containing protein (VCP) mutation in our study and overview of the VCP mutations associated with amyotrophic lateral sclerosis (ALS). **(A)** Sequencing chromatograms of the VCP p.R155C mutation. **(B)** The pedigree of the familial ALS with VCP p.R155C mutation. Arrow indicates the index patient. **(C)** Schematic graph of the VCP protein and overview of the VCP mutations linked to ALS. The locations of mutations are depicted in the mRNA structure where exons are numbered 1–17.

One known heterozygous missense mutation in the VCP gene, c.463C>T (p.R155C) (Figure 1A) was identified in one familial ALS proband (III-6) and his affected sister (III-5) (Figure 1B), as well as another sporadic patient in the cohort of 275 sporadic ALS cases and 15 familial ALS pedigrees. The same VCP p.R155C mutation was detected in the additional patient referred to Fujian Medical University Union Hospital in 2021. No parental DNA samples of patient 3 and patient 4 were available for sequencing. No variants in other ALS-related genes were identified in these patients.

### Clinical Features of Patients With VCP p.R155C Mutation

Clinical features of the four patients carrying VCP p.R155C mutations in this study are summarized in Table 1.

**Table 1:** Clinical features of the amyotrophic lateral sclerosis (ALS) patients with valosin-containing protein (VCP) p.R155C mutation in this study.

**Case**	**Gender**	**Age of**	**Site at**	**Bulbar**	**Phenotype**	**Additional**	**Disease**	**Disease**
		**onset (years)**	**onset**	**symptom**		**symptoms**	**progression (ΔALSFRS-R/m)**	**duration (month)**
Patient 1 III-6	Male	41	Lower limbs	Yes	Classic ALS	No	0.2	>116 (alive)
Patient 2 III-5	Female	51	Lower limbs	No	Classic ALS	No	0.2	>42 (alive)
Patient 3	Male	48	Lower limbs	No	PMA	No	0.2	>102 (alive)
Patient 4	Male	44	Lower limbs	No	PMA	PDB	0.2	>45 (alive)

*ALSFRS-R, revised ALS functional rating scale; PDB, Paget's disease of bone; PMA, progressive muscular atrophy*.

The familial ALS proband (III-6, Patient 1) was a 51-year-old male with progressive weakness in four limbs. At the age of 42 years, he complained of weakness in the lower limbs. He felt clumsy when climbing upstairs and unusual fatigue when walking a long distance. He began to have difficulties in uplifting his arms 4 years later. In the following years, he developed weakness of hands. No cognitive deficits or behavioral changes were noted. The neurologic examination at 7 years after onset revealed obvious weakness and atrophy of four limbs (MRC 4/5), more severe in the proximal muscles of limbs, with diffused reduction of deep tendon reflexes. Babinski's sign was elicited bilaterally. The sensory system and cognition were not affected. Serum creatine kinase (CK) level was normal. Electromyography (EMG) demonstrated fibrillations and positive sharp waves in muscles of four limbs and left thoracic paravertebral muscles, with muscle unit action potentials of increased amplitude, prolonged duration, and reduced recruitment in muscles of limbs. The ALS Functional Rating Scale-Revised (ALSFRS-R) score was 39/48. The score of Montreal Cognitive Assessment (MoCA) testing was 27/30. He developed exertional dyspnea 9 years after onset and began to use the non-invasive positive-pressure ventilation. At the last follow-up at 116 months after onset, he could still walk slowly with help and do daily life activities without assistance. He had neither dysarthria nor dysphagia. A repeated ALSFRS-R score was 30 with an estimated progression rate of 0.2 score/month since symptom onset. The sister of proband (III-5, Patient 2) had paraparesis in the legs since she was 51 years. She only had some trouble running and climbing upstairs. Neurological examination at 2 years after onset revealed mild atrophy and weakness of lower limbs (MRC 4/5). The deep tendon reflexes were brisk in four limbs. Hoffmann's sign and Babinski's sign were elicited bilaterally, with no sensory and bulbar involvement. Serum CK level was normal. EMG revealed acute and chronic neurogenic changes in all limbs and thoracic muscles. The ALSFRS-R score was 46/48. The score of MoCA testing was 28/30. At the last follow-up at 42 months after onset, she could still walk and go upstairs slowly with a cane, without the involvement of upper limbs. The ALSFRS-R score was 41 with an estimated progression rate of 0.2 score/month since symptom onset. Their father (II-2) died of acute myocardial infarction at the age of 72 years without evident symptoms of limb weakness, and their unaffected mother was still alive. The grandfather (I-1) of the proband exhibited signs of weakness in lower limbs and stayed bedridden for over 10 years before he died in his 70s. However, he was not formally diagnosed with ALS.

Patient 3 was a 57-year-old male who noticed weakness in the legs at the age of 48 years. Initially, he only had some difficulties in climbing stairs and standing up from a squatting position. In the following years, the weakness progressed distally to the feet and he began to have troubles in running and long-distance walking. Four years later, the patient was referred to physicians for his difficulty in lifting his arms and aggravated trouble of walking. There were no behavioral symptoms. Neurological examination 5 years after onset revealed obvious atrophy of muscles of arms and lower limbs with fasciculations. Deep tendon reflexes were decreased in all limbs. Palm-chin reflexes and Babinski's sign was not elicited. EMG indicated the pattern of neurogenic changes in muscles of four limbs, such as abnormal spontaneous potentials, motor unit potentials of prolonged duration, increased amplitude, and reduced recruitment. Serum CK level was 298 IU/L (normal value 22–270 IU/L). The biopsy sample of the biceps muscle demonstrated grouped atrophic fibers with both type I and type II fibers. He scored 42/48 on ALSFRS-R and 28/30 on MoCA testing. He began to use wheelchair 7 years after onset. At the last follow-up visit at 102 months after onset, he was bedridden, but still independent for daily life activities, such as writing, dressing, and eating meals, without bulbar involvement. The ALSFRS-R score was 31/48 with an estimated progression rate of 0.2 score/month since symptom onset. His parents were in their 80s and healthy.

Patient 4 presented with weakness of the left leg at the age of 44 years and felt clumsy when climbing upstairs. After 2 years, he developed weakness of the right leg. Muscle atrophy with fasciculation in lower limbs was noticed. Serum CK level was normal. Serum alkaline phosphatase level was 1,532 U/L (normal value 50–135 IU/L). Shoulder, pelvic, and lumbar spine radiographs showed osteolysis, osteosclerosis, and cortical thickening. An isotope bone scan showed increased tracer uptake in the affected bones. He did not suffer from bone pain. He was diagnosed with PDB and treated with zoledronic acid injection, calcitriol, and calcium carbonate. The serum alkaline phosphatase level decreased obviously to about 200 U/L. He gradually developed weakness of both arms and had difficulties lifting arms. Neurological examination at 33 months after onset revealed obvious muscle atrophy of arms and legs with fasciculations. Muscle strength was decreased in the upper limbs (MRC 4/5 in distal limbs and MRC 3/5 in proximal limbs) and the lower limbs (MRC 3/5 in distal limbs and MRC 4/5 in proximal limbs). Deep tendon reflexes were decreased in all limbs. Babinski and Hoffman's signs were not elicited. He was cognitively normal, with a score of 28/30 on MoCA testing. He scored 42/48 on ALSFRS-R. EMG demonstrated chronic and active denervation of the upper and lower limbs, rectus abdominis, and sternocleidomastoid muscles. At the last phone follow-up at 45 months after onset, he could still walk independently for 1 km and do daily life activities, such as writing, dressing, eating meals, and driving, without bulbar involvement. The ALSFRS-R score was 39/48 with an estimated progression rate of 0.2 score/month since symptom onset. Both parents of patient 4 were healthy in their 70s.

### Prevalence of VCP Mutations in Patients With ALS in Different Populations

We identified 44 studies screened VCP mutations in patients with FALS and/or SALS (Table 2). The frequency of VCP mutations is 0.08% (95% *CI* 0.00–1.26%) in patients with FALS and 0.02% (95% *CI* 0.00–0.15%) in patients with SALS in Chinese. The frequency of VCP mutations is 0.37% (95% *CI* 0.00–1.43%) in patients with FALS and 0.09% (95% *CI* 0.00–0.38%) in patients with SALS in Japanese. The frequency of VCP mutations is 0.28% (95% *CI* 0.12–0.52%) in patients with FALS and 0.08% (95% *CI* 0.03–0.15%) in patients with SALS in Caucasian populations. In pooled analysis, the frequency of VCP mutations is 0.28% (95% *CI* 0.12–0.50%) in patients with FALS and 0.06% (95% *CI* 0.02–0.12%) in patients with SALS (Table 2).

**Table 2:** Summary of studies screened VCP variants in patients with ALS.

	**Race (origin)**	**FALS**	**FALS with *VCP* variants**	**SALS**	**SALS with *VCP* variants**
Zou et al. (63)	Chinese	20	0	324	0
Liu et al. (11)	Chinese	20	0	234	0
Pang et al. (12)	Chinese	4	0	46	1
Tsai et al. (13)	Chinese	39	0	216	0
Zhang et al. (14)	Chinese	-	-	311	0
Liu et al. (15)	Chinese	24	0	-	-
Chen et al. (16)	Chinese	15	0	253	0
This study	Chinese	15	1	275	1
Hirano et al. (17)	Japanese	-	-	75	1
Nakamura et al. (18)	Japanese	39	1	469	0
Nishiyama et al. (19)	Japanese	111	0	-	-
Naruse et al. (20)	Japanese	89	1	410	1
Narain et al. (21)	Indian	5	0	149	0
Johnson et al. (5)	Caucasian	215	5	73	0
Williams et al. (22)	Caucasian	131	0	48	0
Abramzon et al. (23)	Caucasian	-	-	701	3
González-Pérez et al. (6)	Israeli-Arab	274	5	178	0
Koppers et al. (24)	Dutch	80	1	1,076	1
Miller et al. (25)	British	75	0	101	0
Tiloca et al. (26)	Italian	166	0	14	0
Kenna et al. (27)	Irish	50	0	389	1
Le Ber et al. (28)	French	-	-	26	0
Couthouis et al. (29)	American	-	-	242	0
McCluskey et al. (30)	American	20	1	-	-
Cady et al. (31)	American	42	1	349	0
Kwok et al. (32)	British	102	2	90	0
Krüger et al. (33)	German	6	0	74	1
Cooper-Knock et al. (34)	British	42	0	-	-
Gibson et al. (35)	American	-	-	87	0
McCann et al. (36)	Australian	212	0	-	-
Morgan et al. (37)	British	131	0	995	1
Türk et al. (38)	German	-	-	43	0
Dols-Icardo et al. (39)	Spainish	10	0	44	1
Lamp et al. (40)	Italian	58	0	210	0
Müller et al. (41)	German	301	1	-	-
Mehta et al. (42)	British	100	0	841	2
Tripolszki et al. (43)	Hungarian	3	0	104	0
Kotan et al. (44)	Turkish	10	1	45	0
Pensato et al. (45)	Italian	34	1	179	3
Ungaro et al. (46)	Italian	66	0	931	0
Yilmaz et al. (47)	German, Swedish	418	0	-	-
McCann et al. (48)	Australian	-	-	616	0
Nunes Gonçalves et al. (49)	Brazilian	93	1	-	-
Shepheard et al. (50)	British	7	0	93	0

*ALS, amyotrophic lateral sclerosis; FALS, familial amyotrophic lateral sclerosis; SALS, sporadic amyotrophic lateral sclerosis*.

### Literature Review of the Phenotype of ALS Patients With VCP Mutations

In addition, 46 VCP mutated ALS patients with detailed clinical features in previous research were identified. The clinical characteristics of these patients are summarized in Table 3. The mean age at onset was 50.29 ± 10.55 years, ranging from 24 to 68 years. Most of the VCP-related patients with ALS were Caucasian (76.1%, 35/46), only 9 cases were of Asian origin (19.6%, 9/46). Thirty-seven patients (80.4%) had a family history of ALS, FTD, dementia, parkinsonism, or PDB, including 31 patients (67.4%) who had a family history of ALS. Thirty-two (69.6%) patients claimed that the weakness started from limbs, and only six 13.0% patients developed a bulbar-onset. Co-occurrence of FTD, PDB, parkinsonism, myopathy, or psychiatric diseases was reported in 18 (39.1%, 18/46) ALS cases carrying VCP mutations.

**Table 3:** Summary of previously published VCP-causing ALS cases with detailed records.

**Base**	**Amino**	**Exon**	**Domain**	**Race**	**Family**	**Gender**	**Age of**	**Site of**	**Phenotype**	**Additional**	**Disease**	**Reference**
**change**	**acid change**			**(origin)**	**history**		**onset (years)**	**onset**	**of ALS**	**symptoms**	**duration**	
c.179A>G	p.K60R	3	CDC48	Italian	No	F	<69	NA	Classic ALS	Cognitive impairment	NA	(45)
c.266G>A	p.R89Q	3	CDC48	Chinese	No	M	24	Limbs	Classic ALS	No	5 months	(7)
c.271A>T	p.N91Y	3	CDC48	Brazilian	Myopathy, FTD	M	36	Limbs	PMA	No	>4 years (alive)	(51)
c.277C>T	p.R93C	3	CDC48	Italian	ALS, PBD, AD	M	47	Lower limbs	Classic ALS	No	>13 years (alive)	(45)
c.293A>T	p.D98V	3	CDC48	Japanese	ALS	M	58	Limbs (proximal right leg, distal right arm)	Classic ALS	FTD	>10 years (alive)	(52)
c.340A>G	p.I114V	4	CDC48	Dutch	Dementia	F	52	Lower limbs (bilateral)	Classic ALS	No	>119 months (alive)	(24)
c.340A>G	p.I114V	4	CDC48	Caucasian	ALS	NA	45	Upper limbs (distal bilateral)	NA	No	27 months	(6)
NA	p.G128A	4	CDC48	Mixed Caucasian	ALS, PDB, PD, myopathy	M	48	NA	NA	No	NA	(53)
c.451A>G	p.I151V	5	CDC48	African- American	No	F	68	Lower limbs	Classic ALS	No	30 months (19 months to AV)	(54)
c.463C>T	p.R155C	5	CDC48	Italian	No	M	42	Limbs	Classic ALS	Myopathy	NA	(45)
c.463C>T	p.R155C	5	CDC48	Italian	ALS	F	29	Upper limb (left hand)	PMA	No	>11 years (alive)	(55)
c.463C>T	p.R155C	5	CDC48	Japanese	ALS, FTD	F	35	Upper limb (proximal right)	Classic ALS	No	5 months to AV, alive	(52)
c.463C>T	p.R155C	5	CDC48	American	ALS, FTD, myopathy, PBD	F	45	Lower limbs (proximal bilateral)	Classic ALS	Myopathy	3 years	(56)
c.464G>A	p.R155H	5	CDC48	Caucasian	ALS, PBD, IBM, parkinsonism, dementia	NA	53	Upper limb (distal left)	NA	No	39 months	(5)
c.464G>A	p.R155H	5	CDC48	Caucasian	FTD, IBM, PBD, PD, psychiatric symptoms	NA	63	Limbs	Classic ALS	No	21 years	(6)
c.466G>T	p.G156C	5	CDC48	Japanese	ALS, psychiatric symptoms	M	51	Lower limbs	Classic ALS	No	>4 years (alive)	(57)
c.466G>T	p.G156C	5	CDC48	Japanese	ALS	F	34	Upper limbs	Classic ALS	Psychiatric symptoms	34 months (8 months to AV)	(57)
c.472A>G	p.M158V	5	CDC48	Japanese	No	M	36	Limbs (right)	NA	PDB	5 years (2 years to AV)	(58)
c.475C>G	p.R159G	5	CDC48	American	ALS, PDB, dementia	NA	53	Lower limbs	Classic ALS	Cognitive impairment	2 years to AV, alive	(5)
c.475C>G	p.R159G	5	CDC48	American	ALS, dementia	NA	46	Lower limbs	NA	PDB	5 years	(5)
c.475C>T	p.R159C	5	CDC48	American	No	F	68	Lower limbs	Classic ALS	No	>5 years (alive)	(23)
c.475C>T	p.R159C	5	CDC48	Caucasian	ALS, PDB	NA	62	NA	Classic ALS	No	24 years	(6)
c.475C>T	p.R159C	5	CDC48	Caucasian	ALS	NA	57	Limbs	Classic ALS	PDB	NA	(6)
c.475C>T	p.R159C	5	CDC48	Caucasian	ALS, PDB	NA	53	NA	Classic ALS	No	16 years	(6)
c.475C>T	p.R159C	5	CDC48	Caucasian	ALS, PDB	NA	53	NA	Classic ALS	No	NA	(6)
c.476G>A	p.R159H	5	CDC48	Dutch	FTD, MS	F	59	Upper limbs (distal bilateral)	Classic ALS	No	23 months	(24)
c.476G>A	p.R159H	5	CDC48	Geek	FTD, dementia, myopathy	M	40s	NA	Classic ALS	No	>3 years (alive)	(59)
c.571C>G	p.R191G	5	Linker 1	Israeli-Arab	ALS, myopathy, parkinsonism	NA	50	Bulbar	Classic ALS	No	11 years	(6)
c.571C>G	p.R191G	5	Linker 1	Israeli-Arab	ALS, myopathy, parkinsonism	NA	42	Bulbar	Classic ALS	Myopathy	9 years to AV, alive	(6)
c.571C>G	p.R191G	5	Linker 1	Israeli-Arab	ALS, myopathy, parkinsonism	NA	<45	Bulbar	Classic ALS	Myopathy, parkinsonism	9 years	(6)
c.571C>G	p.R191G	5	Linker 1	Israeli-Arab	ALS, myopathy, parkinsonism	NA	<45	Bulbar	Classic ALS	Myopathy	NA	(6)
c.571C>G	p.R191G	5	Linker 1	Israeli-Arab	ALS, myopathy, parkinsonism	NA	<45	Bulbar	Classic ALS	Myopathy, parkinsonism	7 years to AV, alive	(6)
c.572G>A	p.R191Q	5	Linker 1	Italian	ALS, FTD/dementia, parkinsonism, PDB	NA	51	Upper limb (proximal right)	Classic ALS	No	29 months (11 months to AV)	(5)
c.572G>A	p.R191Q	5	Linker 1	Italian	ALS, FTD/dementia, parkinsonism, PDB	NA	53	Limbs (left)	Classic ALS	No	2 years to AV, alive	(5)
c.572G>A	p.R191Q	5	Linker 1	Italian	ALS, FTD/dementia, parkinsonism, PDB	NA	50	Right lower limb	Classic ALS	Cognitive impairment	>54 months (alive)	(5)
c.572G>A	p.R191Q	5	Linker 1	Italian	ALS, FTD, dementia, parkinsonism, PDB	NA	37	Lower limbs (distal bilateral)	Classic ALS	No	>4 years (alive)	(5)
c.572G>A	p.R191Q	5	Linker 1	Caucasian	ALS	M	42	Lower limb	Classic ALS	No	>12 years (alive)	(5)
c.572G>A	p.R191Q	5	Linker 1	Japanese	Demyelinating polyneuropathy, IBM	M	53	Lower limbs (distal bilateral)	Classic ALS	No	>1 years (alive)	(52)
c.572G>C	p.R191P	5	Linker 1	Turkish	ALS, FTD	F	60	Lower limb	Classic ALS	FTD	NA	(44)
c.572G>C	p.R191P	5	Linker 1	Turkish	ALS, FTD	F	48	NA	NA	No	NA	(44)
c.572G>C	p.R191P	5	Linker 1	Turkish	ALS, FTD	F	60	NA	Classic ALS	FTD	NA	(44)
c.1160A>C	p.N387T	10	D1	Caucasian	No	M	57	Lower limb	Classic ALS	No	>5 years (alive)	(23)
c.1460G>A	p.R487H	12	D2	Japanese	FTD, PD	M	61	Upper limbs (proximal bilateral)	PMA	Dementia	5 years to AV, alive	(17)
c.1460G>A	p.R487H	12	D2	Japanese	ALS	M	62	Left lower limb	Pyramidal ALS	FTD	78 months	(60)
c.1774G>A	p.D592N	14	D2	Caucasian	ALS	NA	52	Bulbar	Classic ALS	No	<1 year	(5)
c.1984C>T	p.R662C	14	D2	Caucasian	No	M	67	Lower limb	Classic ALS	No	>2 years (alive)	(23)

*ALS, amyotrophic lateral sclerosis; AV, assisted ventilation; FTD, frontotemporal dementia; MS, multiple sclerosis; NA, not available; PD, Parkinson disease; PDB, Paget's disease of bone; IBM, inclusion body myopathy; IMV, invasive mechanical ventilation*.

## Discussion

In the present study, we reported the phenotype of four patients carrying the known VCP p.R155C mutation, such as two siblings from a familial ALS pedigree and a sporadic individual from a Chinese ALS cohort of 275 sporadic and 15 familial ALS pedigrees.

Mutations in the VCP gene have previously been identified in Caucasian patients with ALS (5, 6, 24, 27, 45, 61, 62). Our meta-analysis showed that the presence of VCP mutations was 0.28% (95% *CI* 0.12–0.52%) in FALS and 0.08% (95% *CI* 0.03–0.15%) in patients with SALS in the Caucasian populations. The prevalence of VCP mutations in the Asian populations has not been well determined since the published ALS-VCP cases were mostly of Japanese origin (17, 52, 57, 58, 60). A VCP p.R487H mutation was identified in one out of 75 Japanese patients with SALS (1.3%) (17), and a p.R155C mutation was detected in one out of 39 Japanese FALS pedigrees (2.6%) but no VCP mutations were found in 469 SALS individuals (18). Our meta-analysis showed that the presence of VCP mutations was 0.37% (95% *CI* 0.00–1.43%) in FALS and 0.09% (95% *CI* 0.00–0.38%) in patients with SALS in Japanese (17–20). No VCP mutations have been discovered among Chinese patients with ALS (11, 13–16, 63) except Pang SY et al. reported a p.G157R mutation in one out of 46 SALS (12) and a recent study identified a p.R89Q mutation in one SALS case in a cohort of 27 unrelated young-onset patients with ALS (7). The higher frequency of VCP mutations in FALS (6.7%, 1/15) and SALS (0.4%, 1/275) in our study may be due to the small sample. Our meta-analysis showed that the frequency of VCP mutations is 0.08% (95% *CI* 0.00–1.26%) in FALS and 0.02% (95% *CI* 0.00–0.15%) in SALS in Chinese, which is lower than Japanese and Caucasian populations. The overall pooled mutation frequency of VCP mutations is 0.28% (95% *CI* 0.12–0.50%) in patients with FALS and 0.06% (95% *CI* 0.02–0.12%) in patients with SALS.

Valosin-containing protein p.R155C mutation was first associated with ALS in 2012 (6), before which it was found in patients with IBMPFD (64). The mutation was subsequently observed in two ALS cases without FTD or PBD from a large cohort study of 190 individuals carrying VCP variants (61) and another survey on 36 families with diverse VCP mutants (62). The onset age of the VCP p.R155C mutated patients in our study ranged from 42 to 51 years, consistent with the onset age of four VCP p.R155C-mutated ALS cases reported previously (29, 35, 42, 45, respectively) (Table 3). It seems that patients carrying VCP p.R155C mutations tend to have a young onset. The three patients carrying VCP p.R155C mutation in our study all had a limb-onset. Our system review revealed that 69.6% of VCP-related ALS had limb-onset ALS, and only patients carrying VCP p.R191G and p.D592N mutation had bulbar onset (Table 3). Patient 1 and 2 in our study demonstrated a phenotype of ALS while patient 3 had a phenotype of progressive muscular atrophy (PMA). They all presented with rather symmetrical proximal muscle weakness in the lower limbs at onset and subsequently progressed to distal lower limbs and upper limbs. Patient 4 manifested weakness in the left leg and developed a phenotype of PMA. Interestingly, two VCP p.R155C mutated patients with ALS reported previously also presented with symmetrical involvement of proximal muscles weakness of arms or legs (52, 56). The system review of previous studies showed that patients carrying VCP p.R487H and p.R159G mutation demonstrated the same phenotype (5, 17). The four p.R155C mutation carriers in our study demonstrated a slow progression (ΔALSFRS-R/month = 0.2 score/month) and two had a long survival duration of more than 102 months and 116 months, respectively. The grandfather (I-1) of the proband also exhibited signs of weakness in lower limbs and stayed bedridden for over 10 years before he died in his 70s. An Italian ALS patient with VCP p.R155C mutation reported by Battistini et al. also had a survival of more than 11 years (55). However, a 35-year-old female with p.R155C mutation progressed rapidly and received tracheotomy positive-pressure ventilation within 5 months after onset (52). It is interesting that in our study the father of proband who was supposed to carry the VCP p.R155C mutation had no symptom of muscle weakness and atrophy before he died of acute myocardial infarction at the age of 72 years, indicated incomplete penetrance of VCP p.R155C mutation. Phenotypic variability in VCP p.R155C mutated ALS pedigree has been reported. In an Italian family, the proband with harboring VCP p.R155C mutation developed young-onset ALS with diffuse severe weakness and wasting in the limbs and rimmed vacuoles in muscle biopsy, while his mother and maternal aunt suffered from mild symptoms limited to lower limbs (45). Our system review showed that ALS patients with some VCP mutations (p.R93C, p.D98V, p.I114V, p.R155C, p.R155H, p.R159C, p.R191G, and p.R191Q) had a relatively slow progression and survival of more than 10 years (Table 3), which is consistent with the phenotype of our patients. However, some ALS patients with VCP mutations (p.R89Q, p.I114V, p.I151V, p.R155C, p.G156C, p.R159H, p.R191Q, and p.D592N) developed a rapid progression and had a survival of fewer than 3 years (Table 3). Phenotype variability associated with different or the same VCP mutation suggests the possible role of modifying genes and/or environmental factors. Extensive genetic studies in different populations to identify more ALS patients with VCP mutations may provide more insight into the genotype-phenotype correlations and the diversity of clinical phenotypes of VCP mutations.

Patient 4 carrying VCP p.R155C mutation developed a phenotype of PDB in addition to PMA. A VCP p.G97E mutation was reported in a Chinese family with IBMPFD without ALS (65). Patient 4 in our study was the first VCP mutation carrier manifesting PDB in addition to ALS in the Chinese population. The co-occurrence of FTD, PDB, parkinsonism, myopathy, or psychiatric diseases was commonly seen in the ALS cases carrying VCP mutations (Table 3). The co-existence of FTD or cognitive impairment was found in patients with seven VCP mutations (p.K60R, p.D98V, p.R155C, p.R159G, p.R191Q, p.R191P, and p.R487H) (5, 17, 44, 45, 52, 56, 60), while PDB was diagnosed in ALS patients with six VCP mutations (p.R93C, p.G128A, p.R155C, p.M158V, p.R159G, and p.R159C) (5, 6, 45, 53, 56, 58). Co-occurrence of myopathy was reported in ALS patients with VCP p.R155C and p.R191G mutations (6, 45, 56), and psychiatric disorders were found in patients with p.G156C and p.R159G mutations (5, 57). Parkinsonism is presented in familial ALS cases with VCP p.R191G mutation (6). Increasing evidence has shown that VCP-related disease may be a multisystem proteinopathy that has a wide clinical spectrum of IBM, FTD, PDB, and parkinsonism apart from ALS (66–68). The phenotypic diversity of the same VCP mutation may indicate the possible role of modifying genes and/or environmental factors.

Except for the hexanucleotide expansions and single base-pair substitutions in the 5' UTR or 3' UTR region of VCP predicted as pathogenic without definite experimental evidence (32), 26 mutations in 17 exons of the VCP gene have been identified in patients with ALS (Figure 1C). All mutations are heterozygous missense mutations. Residues of R155, R159, and R191 are three hot spots of VCP mutations of patients with ALS. VCP is divided into 4 domains, such as one N-terminal CDC48 region, two AAA ATPase domains (D1 and D2), and one C-terminal domain. VCP mutations are predominantly located within the N-terminal ubiquitin-binding domain (69.2%, 18/26), indicating that the malfunction of poly-ubiquitinated protein degradation may be the major pathogenesis of ALS (2). Within the ring-shaped VCP hexamer, the N-terminal domain serves as an indispensable binding region for interaction with target co-factor molecules (2). When pathogenic mutations occur in the N-domain of VCP protein, such as p.R155C, p.R159H, p.R95G, p.G97E, and p.A232E, the stress response is impaired resulting in the incorrect translocation of this hexameric ATPase and assembly disorder (69). Besides the aberrant aggregation of transactive response DNA-binding protein of 43 kDa (TDP-43) (5, 70), the nuclear-to-cytoplasmic mislocalization of fused in sarcoma (FUS) protein in motor neurons has been identified as another pathogenic feature of VCP-mutated ALS, which was ascribed to the increased intron retention (IR) in splicing factor proline and glutamine rich (SFPQ) transcripts (71, 72). Using ALS patient-specific induced pluripotent stem cell (iPSC) models, Patani et al. further unveiled that the four abnormal accumulated sequence-specific intron retention transcripts (IRTs) in VCP mutations included SFPQ, OGT, TUSC3, and DDX39, and their binding affinity to RNA binding proteins (RBPs) could be the key attributes for RBP localization (73, 74). In future, further experiments on pathomechanism of VCP-mutated ALS should be conducted on transgenic animal models as well as patient-specific iPSC models.

In summary, we reported the first VCP mutation carrier manifesting ALS with PDB of ALS in the Chinese population. Our findings expand the phenotypic spectrum of VCP mutations in Chinese patients with ALS and suggest that ALS patients with VCP p.R155C mutation tend to present with relatively young onset, symmetrical involvement of proximal muscles weakness of arms or legs, and then progressed to distal muscles of limbs.

## Data Availability Statement

The datasets presented in this study can be found in online repositories. The names of the repository/repositories and accession number(s) can be found at: https://www.ncbi.nlm.nih.gov/, sra/PRJNA791140.

## Ethics Statement

The studies involving human participants were reviewed and approved by Ethics Committee of Fujian Medical Union Hospital and Henan Provincial People's Hospital. The patients/participants provided their written informed consent to participate in this study.

## Author Contributions

Z-YZ and S-YF designed and conceived the study. Z-YZ, S-YF, and HL performed the analysis of mutations in all the patients. HL wrote the manuscript. Z-YZ critically revised the manuscript. All remaining authors participated in the analysis of data, discussion of the final manuscript.

## Funding

This study was supported by grants from the National Natural Science Foundation of China (81671271 and 81974199) and Joint Funds for the Innovation of Science and Technology, Fujian province (2017Y9002).

## Conflict of Interest

The authors declare that the research was conducted in the absence of any commercial or financial relationships that could be construed as a potential conflict of interest.

## Publisher's Note

All claims expressed in this article are solely those of the authors and do not necessarily represent those of their affiliated organizations, or those of the publisher, the editors and the reviewers. Any product that may be evaluated in this article, or claim that may be made by its manufacturer, is not guaranteed or endorsed by the publisher.
